# Purple corn extract (PCE) alleviates cigarette smoke (CS)-induced DNA damage in rodent blood cells by activation of AMPK/Foxo3a/MnSOD pathway

**DOI:** 10.1080/19768354.2021.1883734

**Published:** 2021-02-23

**Authors:** Wan-Sik Kim, Chea-Ha Kim, Jung-Min Lee, Jeong-Ho Jeon, Beom-Goo Kang, Madhuri Shende Warkad, Gozde Inci, Hong-Won Suh, Soon Sung Lim, Sung-Chan Kim, Jaebong Kim, Jae-Yong Lee

**Affiliations:** a Biochemistry, Hallym University College of Medicine, Chuncheon, The Republic of Korea; b Pharmacology, Hallym University College of Medicine, Chuncheon, The Republic of Korea; c Department of Food and Nutrition, Hallym University, College of Natural Science, Chuncheon, The Republic of Korea

**Keywords:** Purple corn extract, cigarette smoke, DNA damage, blood cells, AMPKFOXO3a-MnSOD

## Abstract

Purple corn extract (PCE) is a nutraceutical, an activator of AMPK, and it has antioxidants and anticancer properties. Therefore, PCE could be a candidate for alleviating cigarette smoke (CS)-induced oxidative DNA damage. This study examined whether PCE can have a protective effect on blood cells in an animal model of cigarette smoke (CS)-induced DNA damage. PCE was orally administered to CS-inhaled Spraque-Dawley (SD) rats, followed by the target cells being examined for markers of DNA damage. The study also sought to elucidate the mechanism of PCE action in the PCE treated animals. SD rat inhalation of CS was for once a day for 30 min, repeated for 7 days. PCE was administered orally before CS inhalation. Pretreatment of the animals with oral PCE kept the numbers of white blood cells (WBC) as well as neutrophils (NE), lymphocytes (LY), monocytes (Mo), eosinophils (EO), abd jasophils (BA) from increasing as those were increased in the CS-inhaling SD rats. The amount of phosphorylated γ-H2AX, a DNA damage marker, was assayed in the circulating blood cells collected from the animals and western blot analysis with anti-Foxo3a, p-Foxo3a, p-AMPK, MnSOD antibodies were performed on those cells. PCE protected the circulating blood cells from CS inhalation-induced DNA damage by 44% as assayed by increases in γ-H2AX. PCE also increased the nuclear localization of Foxo3a by 52% over control cells. Mechanistically, PCE appears to efficiently protect various blood cell types from CS-induced DNA damage through removal of ROS via activation of the AMPK/Foxo3a/MnSOD pathway.

## Introduction

Cigarette smoking has been documented to cause oxidative DNA damage in many tissue types including lung, pancreas, sperm and blood. Besides being a major cause of cancer (Vineis et al. 2004) also lead to a spectrum of other illnesses as well such as cardiovascular, chronic obstructive pulmonary and degenerative diseases (Bayard and Jinot 1992). CS contains thousands of toxic chemicals and various harmful agents in forms of free radicals and reactive oxygen species (ROS) (Bayard and Jinot 1992; Norman 1977; Pryor and Stone 1993).

Oxidative DNA damage from ROS plays a major role in the carcinogenicity of CS (Loft et al. 1992; Gackowski et al. 2003) and there are different forms of DNA damage and modification are seen due to CS, including strand breaks, base oxidation, base alkylation, formation of carcinogen-DNA adduct, chromosome aberration and changes in DNA methylation (Weston et al. 1989; Phillips 1997; DeMarini et al. 2008). To assess the extent of CS-induced DNA damage, various methods include DNA comet assays (Speit et al. 2003; Fracasso et al. 2006), measurement of 8-oxodeoxyguanosine (8-oxodG) (Liu et al. 2011; Moktar et al. 2011; Chen et al. 2015), and γ-H2AX (via phosphorylation of the Ser-139 residue of the histone variant H2AX) (Albino et al. 2009; Garcia-Canton et al. 2012), and DNA adduct (Chen et al. 2007; Vadhanam et al. 2012). Assaying γ-H2AX levels and the comet assay have been most frequently used due to their reliability and simplicity.

For protection against CS-induced DNA damage, various natural products have been tested and reported for activity. Examples include resveratrol, shown to reduce CS-induced apoptosis and DNA strand break in animal models (Csiszar et al. 2008); β-cryptoxanthin, a major carotenoid rich in pupmpkins, decreasing 8-hydroxy-2′-deoxyguanosine (8-OHdG) levels in lung tissue samples of the test animals (Liu et al. 2011); fish oil supplementation, reducing the serum 8-OHdG levels in smokers (Ghorbanihaghjo et al. 2013); quercetin/sodium danshensu/baicalein, inhibiting the levels of 8-OHdG for DNA oxidative damage (Chen et al. 2016); and natural sesquiterpene, β-caryophyllene, inhibiting CS-induced micronuclei formation in treated cells (Di Giacomo et al. 2018).

For purple corn extract (PCE), it contains anthocyanins and phenolic compounds that have been shown in numerous studies to have potent antioxidant properties (Wang and Stoner 2008; Zhang et al. 2014). They are also anti-inflammatory (Reddy et al. 2005), anticarcinogenic (Afaq et al. 2005; Chen et al. 2005), and anti-angiogenic (Huang et al. 2006) properties. In addition, these compounds also ameliorate obesity (Tsuda et al. 2003) and diabetes (Hong et al. 2013). Mechanistically, our team has also reported PCE activating AMPK for its anti-diabetic effects (Huang et al. 2015).

The forkhead box O3a (Foxo3a, or FKHRL1) transcription factor is of FOXO family, playing a crucial role in regulating cell cycle arrest, cell death, ROS detoxification, metabolism and longevity (Kops et al. 2002; Libina et al. 2003, Lee et al. 2019). For Foxo3a, it mediates the resistance to oxidative stress via up-regulation of catalase and MnSOD, both associated with detoxification of ROS in the cell (Kops et al. 2002; Nemoto and Finkel 2002; Heo et al. 2016). Foxo3a has been reported to be activated through phosphorylation by an activated AMPK (Greer et al. 2007). We report here that PCE efficiently protected blood cells from CS inhalation-induced DNA damage in SD rats through activation of the AMPK-Foxo3a-MnSOD pathway.

## Methods

### Animals and smoke inhalation

Five week-old healthy male Sprague–Dawley (SD) rats were purchased from Central Experimental Animal (South Korea), and were managed by Hallym University Animal Care System (Hallym protocol 2016-08). All SD rats were stabilized for a week before random distribution to their experimental groups. The SD rats were cared in house cages maintained at 23 ± 1°C, a light/dark cycle of 12 h/12 h, and humidity of 55 ± 1%. The animals were randomly distributed to three groups (8 SD rats in each group); control group without CS inhalation, a group with CS inhalation/administered with H_2_O and a group with CS inhalation/administered with purple corn extract (PCE). PCE was an ethanol extract of *Zea mays var. kculli* (with a total anthocyanin content of 10%), and it was purchased (Zana Export Co., Peru). CS inhalation was performed in a polyacrylate box equipped with a cigarette holder and a pump. Eight SD rats were placed in the CS inhaling box filled with CS and were allowed to inhale CS for 30 min a day and for 7 days. Thirty min before each CS exposure, PCE or water was administered orally to the rat (10 mg/kg/day, 0.3 ml water solution). At the end of the 7 d protocol, body weights of the animals were determined and blood samples were obtained. After sacrifice and dissection, photos of the organs were taken and the weight of each organ was recorded.

### Western blot analysis

Whole blood cell was isolated from SD rat and the red blood cells (RBCs) were depleted using 1 × RBC lysis buffer (Invtrogen) per manufacturer’s protocol. This was done as the nucleus-lacking RBCs interfere with cell counting and DNA damage analysis. Afterwards, the cells were washed three times with PBS and the cell pellets were then resuspended in lysis buffer (50 mM Tris HCl, pH 7.5, 150 mM NaCl, 1% Triton X-100, 0.5% Nonidet P-40, 10 mM N-ethylmaleimide, 0.2 mM Na_3_VO4, and 0.1 mM PMSF) (2 × 10^6^ cells per 500 µl of lysis buffer). The mix was then incubated on ice for 30 min, followed by sonication with three bursts of 30 s duration on ice. The cell debris was removed by 16,000× *g* centrifugation at 4°C for 5 min. The collected supernatants were stored at −70°C to be used for western blot analysis. The protein concentration of the lysates was determined with the BCA protein assay reagent (Thermo Fisher). The cell lysates were resolved either with 10% or 12% gel SDS-PAGE. The separated proteins in gels were transferred onto an Immobilon-P polyvinylidene difluoride membrane (Thermo Fisher). The membrane filter was then blocked in 5% nonfat powdered milk in TBST (50 mM Tris HCl, pH 7.5, 150 mM NaCl, 0.1% Tween 20). The filter was subsequently incubated with anti-Foxo3a (Cell Signaling), anti-phospho-Foxo3a (S413) (Cell Signaling), anti-actin (Sigma-Aldrich), anti-MnSOD (Sigma-Aldrich), anti-AMPK alpha (Cell Signaling), and anti-phospho-AMPK alpha (T172) (Cell Signaling) antibodies in TBST. The filter was then washed with TBST and incubated with goat anti-mouse IgG horseradish peroxidase conjugated antibody (1:10,000 dilution, Cell Signaling). The proteins were visualized with the ECL reagent (Thermo Fisher) according to the manufactures instructions. To confirm the equal loading of proteins in the SDS-PAGE gel lanes, the blots were also probed with an anti-actin antibody (Cell Signaling).

### Biochemical analysis of the sera

Serum levels of triacylglycerol (TG, µg/dl), cholesterol (total cholesterol, µg/dl), alanine aminotransferase (ALT, unit/l) and aspartate aminotransferase (AST, unit/l) were analyzed using commercial kits (981786, 981823, 981656, 981769, and 981771, all from Thermo Electron, Finland) in a Konelab 20XTi Analyzer (Thermo Electron) at the RIC (Regional Innovation Center) of Hallym University (Chuncheon, South Korea).

### Immunofluorescence

To measure the levels of γ-H2AX (a DNA damage marker) and Foxo3a in the RBC-depleted blood samples from SD rats, the cells were first fixed in 3.5% paraformaldehyde on ice for 5 min. They were then treated with the permeabilization solution (−20°C methanol) on ice for 2 min. The samples were then treated with a blocking solution (5% BSA in PBS) with gentle shaking for 1 h. The slides mounted with the blood cells were incubated with anti-γ-H2AX and anti-Foxo3a antibodies on a shaker (in 5% BSA in PBS) for 2 h. This was followed by 1 hr incubation with the secondary detection antibody (incubated in dark in 5% BSA). For γ-H2AX detection, anti-mouse IgG F(ab’)_2_ fragment (Alexa Fluor^®^ 594 conjugate, Cell Signaling, red color) was used. For Foxo3a detection, anti-Mouse IgG F(ab’)_2_ fragment (Alexa Fluor^®^ 488 conjugate, Cell Signaling, green color) was used. The slides were then sealed with a drop of mounting solution and a glass cover. The immunofluorescence-photographs of the stained blood cells were taken using a confocal microscope (LSM719, Carl Zeiss).

### Blood cell analysis

For RBC-depleted blood samples from CS-inhaled PCE-treated SD rats, cell numbers (numbers of cells/µl) for white blood cells (WBC), neutrophils (NE), lymphocytes (LY), monocytes (MO), eosininophils (EO), and basophils (BA) were measured using a blood cell analyzer (Hemavet 950, Drew Scientific) at the RIC (Regional Innovation Center) of Hallym University (Chuncheon, South Korea).

### Statistical analysis

The statistical analysis was carried out by GraphPad Prism Version 4.0 for Windows (GraphPad Software). *P*-values less than 0.05 were considered to indicate statistical significance. All values were expressed as mean ± S.E.M.

## Results

### Biochemical examination of the sera and blood cells obtained from CS-inhaled PCE-treated SD rats

**Figure 1.**
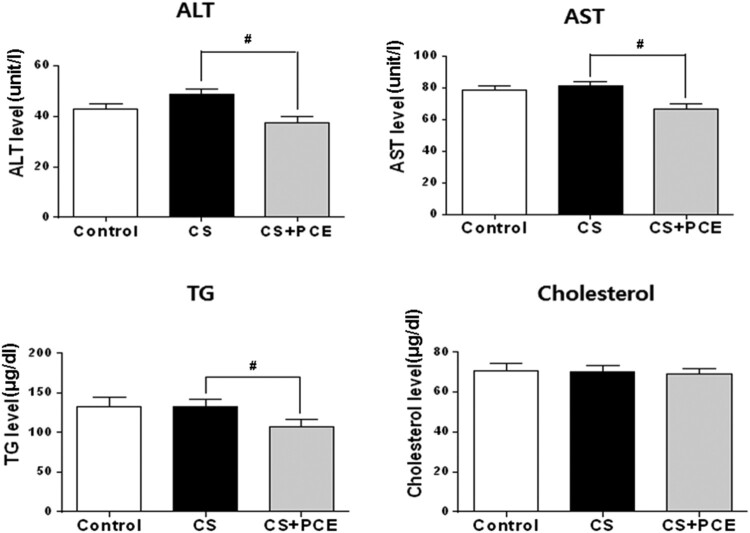
Lipid profile and liver function analysis of CS-inhaled/PCE-treated SD rat serum samples. Levels of AST (aspartate transaminase, unit/l), ALT (alanine transaminase, unit/l), TG (triglyceride, µg/dl), cholesterol (total cholesterol, µg/dl) were measured in sera of CS-inhaled PCE-treated SD rats. The vertical bars indicate the standard error of mean for four different measurements. The CS-inhaled SD rats without PCE treatment (CS) were compared to control SD rats (control) (*P < 0.05, **P < 0.01 and ***P < 0.001) and CS-inhaled/PCE-treated SD rats (CS + PCE) (#*P* < 0.05, ##*P* < 0.01 and ###*P* < 0.001).

**Figure 2:**
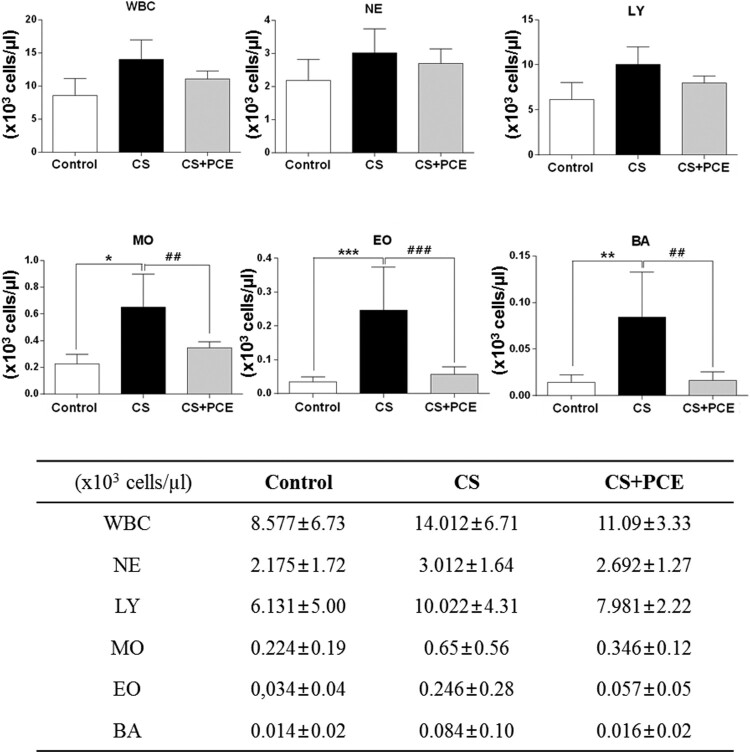
. Analysis of the blood cell type numbers from CS-inhaled/PCE-treated SD rats. (A) Levels (x103 cells/µl) of white blood cell (WBC) as well as neutrophils (NE), lymphocytes (LY), monocytes (MO), eosinophils (EO), and basophils (BA) were measured in the blood (RBC-depleted) obtained from the CS-inhaled/PCE-treated SD rats using a blood cell analyzer. The vertical bars indicate the standard error of the mean from four different experiments. The CS-inhaled SD rats without PCE treatment (CS) were compared to control SD rats (control) (*P < 0.05, **P < 0.01 and ***P < 0.001) and CS-inhaled/PCE-treated SD rats (CS + PCE) (#*P* < 0.05, ##*P* < 0.01 and ###*P* < 0.001).

The CS-inhaled SD rats (CS) when compared to the control SD rats had slightly elevated ALT and AST levels, markers of liver damage (Figure 1), although this was not statistical significant and suggesting that CS inhalation induced slight liver damage. Administration of PCE to the CS-inhaled SD rats (CS + PCE) decreased the levels of AST and ALT when compared to the CS-inhaled SD rats without PCE (CS) (Figure 1). This indicates that the liver damage induced by CS inhalation was protected by PCE administration. PCE did not affect the cholesterol level but showed to decrease the level of triacylglycerol (TG) in blood samples (Figure 1). This appears to be due to AMPK activation by PCE as reported (Huang et al. 2015). The blood cell analysis showed that the number of white blood cell (WBC) as well as neutrophils (NE), lymphocytes (LY), monocytes (MO), eosinophil (EO), and basophils (BA) were increased in the CS inhaled-SD rats as reported earlier (Fernández et al. 2012; Anandha Lakshmi et al. 2014; Shipa et al. 2017). However, for the CS-inhaled/PCE-treated SD rats (CS + PCE), WBC, NE, LY, MO, EO, and BA values remained low and similar to the control (Control) (Figure 2). These results indicate that PCE alleviates the CS inhalation-induced activation of blood cells in SD rats.

### PCE protects against CS inhalation-induced DNA damage in blood cells

**Figure 3.**
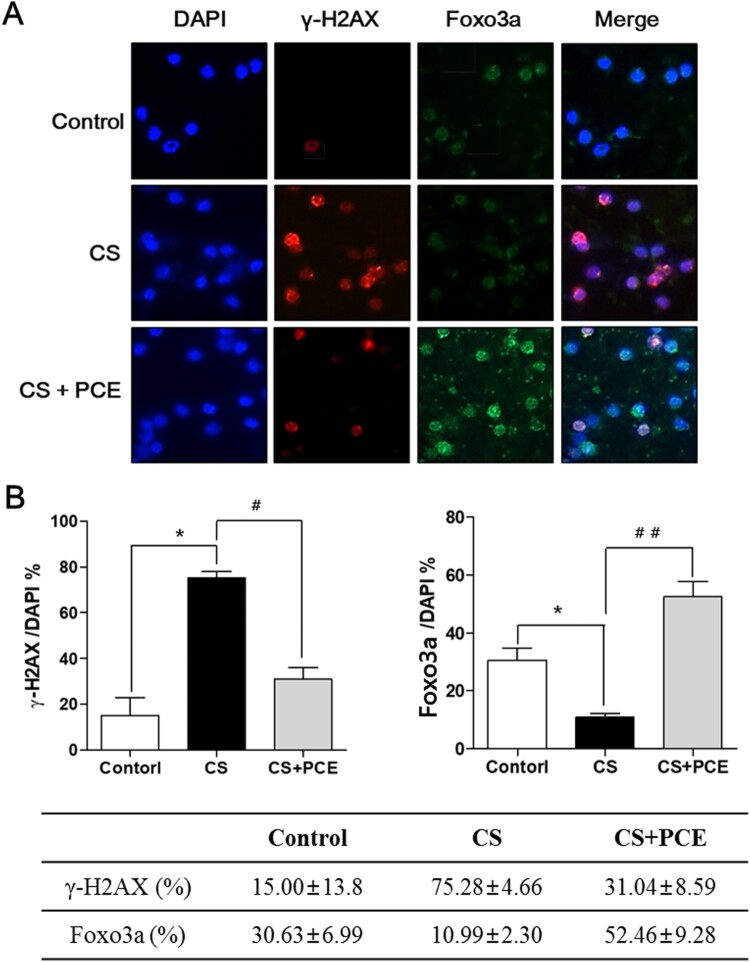
Effect of PCE in CS inhalation-induced DNA damage of the blood cells in SD rats on γ-H2AX and Foxo3a levels. (A) Immunostaining was performed of blood cells (RBC-depleted) obtained from the CS-inhaled PCE-treated SD rats using anti-γ-H2AX and anti-Foxo3a antibody. The anti-mouse IgG, F(ab’)2 fragment (Alexa fluor 594 conjugate, red color) was used as the secondary antibody for γ-H2AX taiing and the anti-mouse IgG, F(ab’)2 fragment (Alexa fluor 488 conjugate, green color) was used as the secondary antibody for Foxo3a staining. DAPI (blue color) was used as the nucleus marker. Cells from the CS-inhaled/PCE-treated SD rats (CS + PCE) were compared to those from the control SD rats (control) and the CS-inhaled SD rats without PCE treatment (CS). (B) Percentages of γ-H2AX-positive and Foxo3a-positive cells among DAPI-stained cells were calculated from Fig. 3A and the data were plotted as bars. The vertical bars indicate the standard error of the mean for four different experiments. The CS-inhaled SD rats without PCE treatment (CS) were compared to control SD rats (control) (*P < 0.05, **P < 0.01 and ***P < 0.001) and CS-inhaled/PCE-treated SD rats (CS + PCE) (#*P* < 0.05, ##*P* < 0.01 and ###*P* < 0.001).

In order to examine the CS inhaling-induced DNA damage of blood cells in SD rats, an immunofluorescence expression profiling for two markers (γ-H2AX and Foxo3a) was performed on RBC (red blood cell)-depleted blood cells from treated SD rats (Figure 3). The blood cells isolated from the CS-inhaled/PCE-treated SD rats were stained with fluorescent-labeled anti-γ-H2AX (red color) and anti-FOPXO3a (green color) antibodies. DAPI was used for nucleus staining (blue color). In this analysis, the level of staining for γ-H2AX-positive blood cells was increased to 75% in the CS-inhaled SD rats when compared to that of the control SD rats (15%) (Figure 3A,B). However, the level of γ-H2AX staining-positive cells was decreased to 31% in the CS-inhaled/PCE-treated SD arts (Figure 3A,B). These results indicate that the CS inhalation induces severe DNA damage in blood cells (increase of 60% over control group) and the PCE administration efficiently protects the cells from the CS inhalation-induced DNA damage in blood cells (by 44% compared with the untreated, CS-exposed group). For the same samples, the level of nuclear localization of Foxo3a, a transcription factor, was decreased in the CS-inhaled SD rats (staining at 10%) when compared to the control SD rat (staining at 30%) (Figure 3A,B). The PCE treatment of CS-inhaled SD rats increased the level of nuclear localization of Foxo3a (up to 52%) (Figure 3A,B), indicating that the increased level of Foxo3a nuclear localization induced by PCE mediates the decrease in CS inhalation-induced DNA damage.

### PCE treatment decreased the CS inhalation-induced DNA damage by activation of AMPK-Foxo3a-MnSOD pathway in the blood cells of SD rats

**Figure 4:**
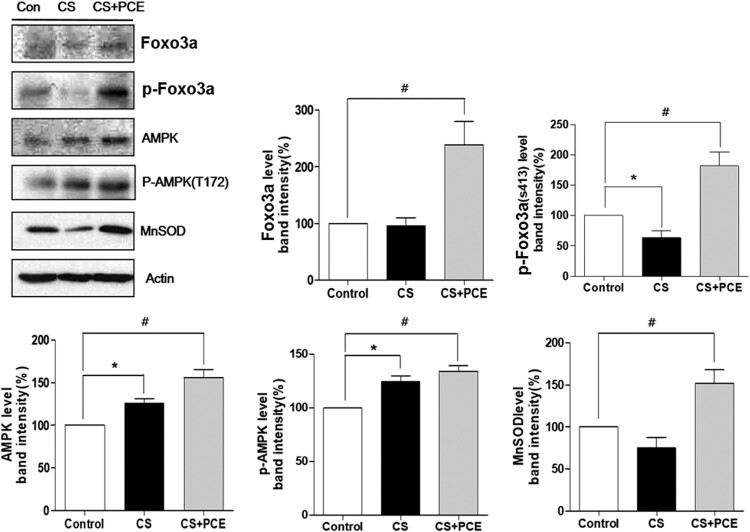
. Effect of PCE on pathway targets for blood cells in the CS inhaled/PCE-treated SD rats. Cell extracts were prepared from blood cells of CS-inhaled/PCE-treated SD rats. The western blot analysis was performed using the cell extracts with anti-Foxo3a, p-Foxo3a (S413), p-AMPK (T172), MnSOD and actin antibodies. Four different western blot analysis results were scanned using A densitometer and the scanned results were plotted as bars. The vertical bars indicate the standard error of the mean for three different experiments. The control SD rats (control) were compared to CS-inhaled SD rats without PCE treatment (CS) (*P < 0.05, **P < 0.01 and ***P < 0.001) and CS-inhaled/PCE-treated SD rats (CS + PCE) (#*P* < 0.05, ##*P* < 0.01 and ###*P* < 0.001).

To elucidate the underlying mechanism for PCE-mediated protection from CS inhalation-induced DNA damage, cell extracts were prepared from rat blood and were subjected to western blot analysis with anti-Foxo3a, p-Foxo3a (S413), p-AMPK (T172), MnSOD and actin antibodies. The results showed that PCE treatment increases the protein levels of p-AMPK (activated AMPK) and Foxo3a. The protein levels for p-Foxo3a (S413) were also increased as a result of direct phosphorylation by p-AMPK. The protein levels for MnSOD, a transcriptional target gene of Foxo3a, were also increased (Figure 4). These results indicate that the PCE-mediated protection from CS inhalation-induced DNA damage appears to result from sequestering of CS-induced ROS via activation of the AMPK/Foxo3a/MnSOD pathway.

## Discussion

It has been well documented that cigarette smoking results in severe DNA damage in various tissues and that many natural compounds can protect the affected tissues from this damage. However, the mechanisms for how certain natural compounds protect against CS-induced DNA damage have not been fully elaborated. In the case of PCE, for this study in SD rats, it showed excellent protection from CS inhalation-induced DNA damage in various blood cells. PCE treated animals had activated levels of AMPK (p-AMPK) which in turn activated Foxo3a (p-S413 Foxo3a), with the resulting p-Foxo3a moving into the nucleus in the cell and activating its transcriptional target, namely MnSOD. The DNA damage in blood cells from CS inhalation-induced ROS appears to have been decreased by induction of MnSOD. Catalase which is also known to be a target gene for Foxo3a is also induced by PCE (Alcendor et al. 2007). Catalase appears to be involved in removal of hydrogen peroxide that is generated via action of MnSOD. CS inhalation increased slightly the protein levels of AMPK and p-AMPK but decreased a little the protein levels of p-Foxo3a and MnSOD (Figure 4). Currently, we cannot explain this discrepancy in expression levels induced by CS. CS inhalation appeared to involve very complicated reactions.

Based on the above findings, PCE could be a good preventive drug candidate for reducing DNA damage due to smoking and the related diseases brought on by this damage including cancer and chronic obstructive pulmonary disease (COPD). In this study, PCE was administered orally to SD rats 30 min prior to daily CS inhalation exposure (30 min in duration). Single dosing of PCE may not be enough to transcriptionally induce sufficient levels of MnSOD. However a continuous daily dosing of PCE for 7 days in this study was adequate to induce effective levels of such target genes involved in reducing oxidative damage from CS-induced ROS.

A number of natural products such as fish oil (Ghorbanihaghjo et al. 2013), andrographolide (Guan et al. 2013), oroxylin A (Li et al. 2016), and caryophyllane (Di Giacomo et al. 2018) have been shown to relieve CS inhalation-induced DNA damage. The protection mechanisms of such agents have been reported to be via activation of p-STAT3, NF-kB or Nrf2 (Li et al. 2016; Lee et al. 2016; Sakurai et al. 2018). The actual mechanisms of protection from CS-induced DNA damage for each of these natural compounds could be different as these are chemicals of different classes and they may also be acting differently on target cells although STAT-3, NF-kB and Nrf2 are known to be involved in the reduction of oxidative stress. Therefore, a characterization of possible relationship between changes in Foxo3a, ROS, STAT-3, NF-kB and Nrf2 levels in blood cells of CS exposed rats would give a better understanding of the detailed mechanisms involved. In addition, a DNA repair-related gene like GADD45 is also known to be a Foxo3a target gene (Tran et al. 2002), and activation of GADD45 via Foxo3a downstream of PCE may also contribute to reduction of DNA damage by increasing the DNA repair activity. However, this has not been experimentally tested, although our unpublished results suggest that several DNA repair factors are Foxo3a transcriptional target genes. A further characterization of the relationship between these DNA repair factors and Foxo3a may provide more complete explanation of the protection mechanisms provided by PCE.
